# Care-Seeking Pattern among Persons with Depression and Anxiety: A Population-Based Study in Sweden

**DOI:** 10.1155/2012/895425

**Published:** 2012-05-10

**Authors:** Anna Wallerblad, Jette Möller, Yvonne Forsell

**Affiliations:** Division of Public Health Epidemiology, Department of Public Health Sciences, Karolinska Institutet, Norrbacka Plan 7, 17176 Stockholm, Sweden

## Abstract

*Background*. In primary care, a vast majority of patients affected with depression and anxiety present with somatic symptoms. Detection rate of psychiatric symptoms is low, and knowledge of factors influencing care seeking in persons affected by depressive and anxiety disorders on a population level is limited. *Objective*. This study aims to describe if persons, affected by depression and anxiety disorders, seek care and which type of care they seek as well as factors associated with care seeking. *Method*. Data derives from a longitudinal population-based study of mental health conducted in the Stockholm County in 1998–2010 and the present study includes 8387 subjects. Definitions of anxiety and depressive disorders were made according to DSM-IV criteria, including research criteria, using validated diagnostic scales. 2026 persons (24%) fulfilled the criteria for any depressive or anxiety disorder. *Results*. Forty-seven percent of those affected by depression and/or anxiety had been seeking care for psychological symptoms within the last year. A major finding was that seeking care for psychological symptoms was associated with having treatment for somatic problems. *Conclusions*. As a general practitioner, it is of great importance to increase awareness of mild mental illness, especially among groups that might be less expected to be affected.

## 1. Introduction

Mental health problems, such as depression and anxiety disorders, are often underrecognized and untreated. Bijl et al. [1] showed that the prospect of being treated increases with the severity of the illness, but also that half of those affected by a serious mental illness remained untreated. It is easy to understand that a serious condition needs treatment to avoid complications such as suicide, need of inpatient care, and disability. However, studies have shown that the risk of such complications did not differ significantly between mild forms of mental illness compared to moderate forms [2]. In several studies, around half of those affected by psychological distress or psychiatric diagnoses had not been seeking care [3–9]. However, even if they seek, the detection rate of psychiatric symptoms is low. A recent meta-analysis of studies regarding general practitioners ability to recognize mild depression showed a detection sensitivity of 56.5% [10]. This emphasizes the importance of further increasing the awareness of mild cases of mental illness. 

In primary care, a vast majority of patients affected by depression and anxiety present with somatic symptoms [11, 12]. Somatic complaints include changes in appetite and libido, lack of energy, sleep disturbances, dizziness, palpitations, dyspnoea, and general aches, and pains such as headache, back and other musculoskeletal pain, and gastrointestinal disturbances.

Identifying persons affected by mental illness, but seeking care for somatic symptoms, is a major difficulty especially in the primary care setting, due to both patient-related issues as well as physician-related issues [13]. It is of importance that somatic symptoms associated with mental health disorders are not confused with somatoform disorders (i.e., conversion, somatization, hypochondriasis, and somatization disorder).

The knowledge of factors influencing care seeking in persons affected by depressive and anxiety disorders in the population is limited. Hence, it is important to elucidate factors associated with care seeking in these groups, over all, and factors associated with not seeking care for psychological symptoms. Knowledge about factors associated with seeking care could support early identification.

## 2. Objectives

This study aims to describe the prevalence of care seeking among persons with depression and anxiety disorders using data from a population-based study in Sweden. First, we aim to study whether affected persons seek care and if care seeking is associated with socioeconomic factors and health status. Further, we aim to study if those who seek care for psychological symptoms at the general practitioners differ compared to those who seek care from other health care facilities or do not seek at all.

## 3. Material and Methods

### 3.1. Study Sample

This study is based on the PART study (an acronym in Swedish for Mental ill-health, Work, and Relationships). PART is a longitudinal population-based study of mental health conducted in the Stockholm County, Sweden. In 1998-1999, 19742 randomly selected Swedish citizens aged 20–64 years, residing in the Stockholm County, were invited to participate and 10441 persons (response rate 53%) responded to the self-administrated questionnaire (baseline) that included questions on demographic and socioeconomic characteristics, somatic and psychiatric health, and use of drugs. Three years after they had answered the first questionnaire (baseline) those who answered were reassessed with another similar questionnaire including questions on health care seeking; 8700 persons participated (retention rate 83%). Both data collections were supplemented with interviews in a subgroup of the respondents. Psychiatrists performed interviews using Schedules for Clinical Assessment in Neuropsychiatry (SCAN) [14], in order to validate the answers of the questionnaires. A comparison between depressions according to the Major Depression Inventory (MDI) used in the questionnaire and SCAN showed good compliance [15]. Nonparticipation analysis, using national registers, performed after the first two waves, revealed that the association between gender, age, income, education, country of birth, and psychiatric diagnoses in the national registers was similar among participants and nonparticipants [16, 17]. For detailed information about the PART study see the technical report [18].

For the purpose of this study we restricted our analyses to the 8387 subjects that participated in both baseline and the first followup, with information on symptoms of depression and anxiety.

### 3.2. Psychiatric Disorders

Definitions of anxiety and depressive disorders were made according to DSM-IV criteria, including research criteria, using validated diagnostic scales based on the questionnaire. The included scales were the Sheehan Patient-Rated (Panic) Anxiety Scale [19] and the Major (ICD-10) Depression Inventory, MDI [20]. Social phobia was assessed using the avoidance part of an instrument developed by Marks and Mathews [21] and for obsessive-compulsive disorders screening questions suggested by the Swedish Psychiatric Association and Swedish Institute for Health Services Development [22] were used. *Anxiety disorders* included panic syndrome with agoraphobia, agoraphobia without panic syndrome, social phobia, obsessive-compulsive disorder, panic syndrome without agoraphobia, anxiety syndrome due to somatic cause, specific phobia, posttraumatic stress syndrome, general anxiety disorder, and acute stress syndrome. *Depressive disorders* included major depressive disorder, dysthymia, and minor depressive disorder. Some of the persons affected by major depressive disorder may have a bipolar disorder since there was no scale for manic episodes in the questionnaire. Three mutually exclusive groups were created: any depressive disorder (*n* = 465), any anxiety disorder (*n* = 751), and coexistent depressive and anxiety disorder (*n* = 810). In total 2 026 persons (24%) fulfilled the criteria for any depressive or anxiety disorder. This corresponds well to other studies [7, 23–25].

### 3.3. Care Seeking

The Swedish health care system is mainly taxpayer funded and largely decentralized. Health care is accessible to everyone living in Sweden, and because of tax subsidies, costs are limited for individuals. Both private- and public-funded outpatient clinics are under the same regulations and the patient can choose their preference for the same cost, with exception for those private clinics without affiliation to the public health care system. With regards to psychologists and psychotherapists, there are also private practices without affiliation, and thus not subsidized, a more expensive alternative for the patient. When it comes to alternative care, it is always to a nonsubsidized cost. The health care system is organized with a broad base of easy-accessible primary care in health centers, where a variety of health professionals (doctors, nurses, physiotherapists, psychologists, counsellors, and other staff members) work. The usual path to seek care is to turn to the health centre to see a specialist in general medicine (General Practitioner, GP). The major part of patients is taken care of at this level, but in case the patient needs to see another specialist, he or she is referred by the GP. The GP can also refer the patient to a psychologist or likewise. Within the psychiatric sector, it is also possible to directly take contact with an outpatient psychiatric clinic, if it is obvious that the mental problems are severe enough to belong to the psychiatric care. If not, the patient will be redirected to the primary health care centre.

Care seeking was evaluated using two questions based on the questionnaire. The first was “Have you, due to sleeping problems, personal problems or psychological symptoms, been in contact with one or more of the following during the last 12 months?” The following response alternatives were given: “psychiatrist public or private,” “psychologist/psychotherapist public or private,” “general practitioner public or private,” “other medical/psychological treatment,” and “alternative medical treatment.” Seeking care for psychological symptoms was defined as having checked one or more of the response alternatives. The second question was “Have you, due to bodily symptoms or somatic illness, been in contact with one or more of the following during the last 12 months?” with the following response alternatives: “general practitioner public or private,” “specialist public or private,” “other medical treatment,” and “alternative medical treatment.” Seeking care for somatic symptoms was defined as having checked one or more alternatives. Multiple responses were possible for both questions.

### 3.4. Characteristics

Data on *country of origin *and *education* was derived from the baseline questionnaire, and all other data was retrieved from the followup.

*Hazardous alcohol* use was evaluated using AUDIT (Alcohol Use Disorders Identification Test) [26, 27]. The cut-off ≥ 8 was used for men and ≥6 for women [28]. Education was categorized into three groups: basic compulsory education (≤9 years), upper secondary education (10–12 years), and higher education (college/university, ≥13 years). Data from the second wave included *household composition, *children in household (permanently or more than half of the time were considered as living with children). *Labour market position *included employment/own business, on leave (studies and parental leave), unemployed or in labour market policy measures, disability pension or sick leave for more than a month, and retirement. *Having a close friend* included the answers entirely or fairly true to the question if there was a special person the person felt he/she could get support from. *Disability last 30 days* included those who had been so affected by psychological symptoms/problems that they had not been able at all to pursue the ordinary tasks. *Somatic illness* was measured by a list of 26 somatic disorders, and only those currently treated by a doctor were considered as exposed to somatic illness.

### 3.5. Statistical Methods

The statistical analyses aimed to describe presence of care seeking and possible factors associated with such among persons affected with depression or anxiety disorders. Also, the analyses describe what factors could be associated with seeking different types of care. This was done by using cross-tabulation in IBM SPSS Statistics 19.0 on different kinds of care seeking to describe the prevalence of care seeking by demographic, socioeconomic, and psychiatric factors. Pearson chi-square tests were used to test for statistical significance. Additionally, to analyse differences between persons seeking different kinds of care, one-way analysis and Bonferoni tests were used. Partially missing answers were treated as missing values in the analyses (varying from 0.04% on born abroad to at most 2.4% for seeking somatic care).

## 4. Results

A description of the study sample, stratified by depressive and/or anxiety disorders, is presented in Table 1. Persons with *depression* were more often female, young, single, living without children, less often having a close friend and less educated. They reported more often to be on sick leave/disability pension, unemployed, treated for somatic illness, having hazardous alcohol use and were more often affected by disability, compared with those without depression and anxiety. Persons with *anxiety* were more often female, younger and more often had hazardous alcohol use, compared with those without depression and anxiety. Persons with comorbid *depression and anxiety* showed similar differences as those affected by depression and also reported more often having country of origin outside Sweden.

### 4.1. Care Seeking for Psychological Symptoms

Of those affected by depression and/or anxiety, 47.1% of the persons stated that they had been in contact with some type of health care facility within the last year due to psychological symptoms; see Table 2. Persons who had been seeking help for psychological symptoms were more often female, older, singles, born abroad, or outside the labour market. Additionally, they more often had comorbidity factors such as somatic illness, or both depression and anxiety, were more severely affected by depression and more often disabled due to psychological symptoms.

When it comes to type of care, 30.4% of the persons affected with depression and/or anxiety had been seeking help for their psychological symptoms at a GP, and 33.5% had been seeking help at other caregivers; see Table 3. About thirteen percent had reported a GP as their only care provider. 

In the group that went to the GP, there was an overrepresentation of persons with both depression and anxiety, and disability due to psychological symptoms as well as somatic illness compared to those that did not seek care. This was applicable for both those who had the GP as their only provider, as well as those who also had seen a psychiatrist, psychologist, or other (alternative/other medical or psychological). Seeking care to a greater extent to psychiatrists or psychologists was also the case for those with comorbid anxiety and depression, and disability due to psychological symptoms. When it came to those with hazardous alcohol use, there was an overrepresentation among those who had seen both a GP and a psychiatrist/psychologist, compared to the persons that had only attended GP or had not been seeking at all.

Those that had been seeking GP were also older and more often they had less education, than those who were more likely to not seek care at all, or to seek only a psychiatrist or psychologist. At the GPs, persons outside the labour market, on sick leave or disability pension were overrepresented, as well as persons born in another country, both among those who had the GP as their only care provider or in combinations. Persons born abroad were also less likely to only have a psychiatrist or psychologist as their only provider.

Regarding the group that turn only to alternative care or other medical/psychological treatment, there seemed to be no differences among groups except for persons born abroad and less educated persons that were underrepresented. 

## 5. Discussion

In the present study we found that 52.5% of those affected by depression and/or anxiety disorders did not seek care for psychological symptoms. Among those not seeking care for psychological symptoms, two-thirds had sought care for somatic symptoms. One reason for seeking for somatic symptoms might be that they primarily have identified the somatic symptoms that often accompanies depression and anxiety, which has been reported in several previous studies [12, 29, 30]. One-third of the affected had not been seeking care at all. Comparison with other studies is somewhat difficult due to different measures on both mental health and of outcomes such as care seeking or treatment. In our study, the proportion seeking care was 47.1%. Several other studies have showed prevalence for seeking care for psychological distress or a variety of psychiatric diagnoses (such as depression, dysthymia, GAD, panic disorder, phobias), ranging from 36 to 60% [3–9]. This shows that the problem with people in need who does not seek help is widely spread.

In the present study, persons less likely to seek help were male, younger, born in Sweden, living with a partner, employed/on leave for studies or parental leave, retired or had higher education. Several studies have reported that prejudices in the general population against male persons affected by mental disorders are higher than against affected females [31]. This might make men less prone to identify their psychological symptoms. Having a job, being a student, or on parental leave might imply less daytime available in to be spent seeking care. When it comes to labour market position, being on sick leave might be a promoting factor but also a result of care seeking per se and an indicator of severity. 

Summarizing, in the group of persons less likely to seek there is an overrepresentation of individuals that perhaps are less likely to be suspected of being affected of mental problems due to lower load of risk factors as well as assumed to be well adjusted in society.

Those with milder symptoms and less disability due to psychological symptoms were also less likely to seek. Evidently this could be due to less need of care, and it could be argued that minor depression and distress could be resolved without professional help [32, 33]. However, mild disorders are increasingly considered clinically significant [34] and detecting them in an early stage might prevent them from turning into serious cases in the future [2, 35, 36].

Having been seeking care both at the GPs and at the psychiatrists or psychologist/psychotherapists could mean, with regards to how the health care system is organized in Sweden, that the GP has referred the patient. This is especially the case when it comes to persons with a hazardous alcohol use that more often had been seeing a GP and a psychiatrist/psychologist. In this study we lacked information on type of clinical specialization but it is likely that it was referrals from GPs to clinics specializing in alcohol dependence.

There was no gender difference for the category that had seen a GP and a psychiatrist/psychologist/therapist. This could possibly stand for that there is no gender difference when it comes to proportional referrals from the GP, which is gratifying.

Persons with higher education were less likely to seek care at all, and if they did, they were more likely to turn to a psychiatrist/psychologist. This could possibly stand for a perceived need for a more specific treatment, higher ability to interpret their symptoms as psychological, or more knowledge on possible places to go. That persons under the age of 35 years show the same pattern could maybe stand for partly the same, as in a perceived need for a more specific treatment, but perhaps also for less stigmata surrounding mental problems. The opposite is shown for persons born abroad; characteristics of migrants' pathways to psychiatric care have been reported to be delays in seeking professional help, a lower probability of medical referral, frequent involvement of the police and emergency services, and high proportions of compulsory and secure-unit admissions [37].

Persons on sick leave, with a disability pension, or unemployed were more likely to see a GP, alone or in combination with psychiatrist/psychologist or other and more likely to seek care. It could be argued that having a long-term psychological health problem might be preceding poorer social functioning resulting in unemployment or sick leave/disability pension. Also, contact with a GP or a psychiatrist is a necessity for the medical certificates needed for the social insurance system initiating a sick leave or disability pension, which in part could explain the overrepresentation among these groups. But also, it could stand for a more severe psychological health status or a greater need of treatment. Studies have shown that unemployment [38], as well as sick leave or disability pension per se, can have a negative effect on psychological health [39].

An important factor for not seeking care for psychological symptoms seems to be not having any treatment for somatic illness. This could be an important finding; if a person has treatment for any somatic problems, he or she already established a relationship to the physician or care-giving facility, which might make bringing up psychological problems easier. Older people might also have an easier access to care due to a prior relationship with their GP based on somatic illness or plainly longer experience of care seeking.

The category turning only to alternative care seemed to have less to do with the mental illness per se, not varying with severity of illness or disability, but instead with socioeconomic factors that could be argued possibly related to limitations such as high cost or less knowledge of such.

## 6. Study Strengths and Limitations

In the present population-based study, validated diagnostic scales for assessing anxiety and depression were used [40–42].

One limitation is the cross-sectional design, which limits the possibilities to draw causal conclusions. The self-reported care seeking was measured retrospectively one year back from filling in the questionnaire. The scales measuring symptoms of depression cover the last 14 days and for anxiety the last 30 days, respectively. It could therefore be argued that persons might have symptoms but not yet contacted health care or that persons might fall out of the depression and/or anxiety group population because they have had symptoms previously but not during the last month. However, when examining reports of the duration of symptoms, we found that, of those having any form of depression, one-third had it more than two years, one-third since more than six months, and one-third between two weeks and six months. Among those with anxiety, all had had symptoms for more than a month according to the used scale.

## 7. Conclusions

As a general practitioner, it is of great importance to further increase awareness of mild cases of mental illness, especially among groups that might be less expected to be affected by mental illness.

## Figures and Tables

**Table 1:** Description of the study sample and stratified by depressive or anxiety disorder status (*n* = 8387).

	All (*n* = 8387)	No depression and/or anxiety (*n* = 6361)	Depression (*n* = 465)	Anxiety (*n* = 751)	Depression and anxiety (*n* = 810)
	%	%	%	%	%
*Gender*					
Male	42.4	45.3	33.8	38.2	28.8
Female	57.6	54.7	66.2	61.8	71.2
*Age*					
23–35 years	28.7	26,9	36.4	34.4	33.0
36–55 years	44.8	44.7	42.7	44.8	47.9
56–68 years	26.4	28,4	20.9	20.8	19.1
Median	45 years	46 years	40 years	43 years	43 years
*Born abroad*	9.3	8.7	10.3	10.7	12.8
*Household composition*					
Living with partner	67.7	70.5	54.8	66.2	54.1
Living with parents	2.4	2.4	1.9	2.3	2.8
Living with other	2.2	2.0	2.4	3.5	2.6
Single	27.7	25.0	40.9	28.1	40.5
*Children in household*	43.0	43.6	38.7	43.1	40.9
*Education*					
Basic compulsory education or less	15.5	14.8	19.8	13.3	20.6
Secondary school	40.0	39.9	41.3	39.7	39.6
University or college	44.5	45.3	38.9	47.0	39.8
*Labour market position*					
Employment/self-employed/on leave/studies/parental leave	85.7	87.2	80.9	87.7	76.2
Unemployment/labour market policy measures	2.7	2.1	3.9	2.9	6.8
Retirement pension	6.1	6.9	4.1	3.3	3.3
Sick leave/disability pension	4.4	2.9	10.1	5.2	12.6
Other	0.8	0.8	0.9	0.7	1.0
*Close friendship*	94.4	95.9	89.7	93.9	86.4
*Somatic illness*	32.0	28.6	42.4	36.4	48.3
*Hazardous alcohol use*	19.6	16.3	29.2	26.7	33.8
*Depression severity*					
Minor depression	3.5		37.2		18.3
Major depression	2.1		62.8		33.6
*Disability last 30 days due to psychological symptoms*	7.6	3.1	19.5	8.4	35.0

All differences within each variable showed significance when tested with chi-square.

**Table 2:** Proportion care seeking for psychological symptoms, among persons affected by depression and/or anxiety (*n* = 959).

	Proportion seeking care for psychological symptoms		*P* value*
	%	*n*	
*Disorder*			
Depression	40.9	190	0.000
Anxiety	36.8	276	
Depression and anxiety	60.9	493	
*Gender*			0.01
Male	43.3	293	
Female	49.4	666	
*Age*			
23–35 years	41.7	288	0.000
36–55 years	47.9	440	
56–68 years	54.9	223	
*Born in Sweden*			0.033
Yes	46.5	833	
No	53.9	125	
*Household composition*			0.000
Living with partner	41.8	497	
Living with parents	46.9	23	
Living with other	46.6	27	
Single	56.5	412	
*Children in household*			0.054
Yes	44.3	370	
No	49.5	587	
*Education*			0.004
Basic compulsory education or less	54.0	194	
Secondary school	43.6	354	
University or college	48.0	411	
*Labour market position*			0.000
Employment/self-employed/on leave/studies/parental leave	42.9	709	
Unemployment/labour market policy measures	61.1	58	
Retirement pension	45.1	32	
Sick leave/disability pension	79.8	150	
Other	52.9	9	
*Close friendship*			0.452
Having a close friend	46.9	855	
Not having a close friend	51.2	103	
*Somatic illness*			0.000
Currently treated	59.6	513	
Currently not treated	38.3	446	
*Hazardous alcohol use*			0.159
Yes	48.4	268	
No	44.8	579	
*Depression severity*			0.001
Minor depression	37.3	109	
Major depression	46.8	81	
*Disability last 30 days due to psychological symptoms*			0.000
Yes	69.2	301	
No	41.3	653	

*P value for chi-square testing.

**Table 3:** Proportion care-seeking among persons with depression and/or anxiety by combination of health care units (*n* = 2005). Only those having complete information on the care seeking questions were included.

	Only GP *n* = 270	GP and psychiatrist/psychologist (*n* = 169)	GP and other *n* = 170	Psychiatrist/psychologist (*n* = 242)	Only other (*n* = 91)	No care seeking *n* = 1063
	*% * (*n*)					
*Diagnosis*						
Depression (459)	13.9 (64)	5.9 (27)	6.3 (29)	10.2 (47)	4.1 (19)	59.5 (273)*
Anxiety (746)	11.4 (85)	5.2 (39)	5.4 (40)	9.7 (72)	5.0 (37)	63.4 (473)*
Depression and anxiety (800)	15.1 (121)*	12.9 (103)*	12.6 (101)*	15.4 (123)*	4.4 (35)	39.6 (317)
*Gender*						
Male (668)	12.7 (85)	7.6 (51)	7.0 (47)	11.4 (76)	4.0 (27)	57.2 (382)*
Female (1337)	13.8 (185)	8.8 (118)	9.2 (123)*	12.4 (166)	4.8 (64)	50.9 (681)
*Age*						
23–35 years (683)	9.1 (62)	7.0 (48)	6.0 (41)	14.6 (100)*	4.8 (33)	58.4 (399)*
36–55 years (909)	12.8 (116)	9.0 (82)	8.9 (81)	12.5 (114)	4.3 (39)	52.5 (477)
56–68 years (354)	22.6 (80)*	9.0 (32)*	11.9 (42)*	7.6 (27)	4.8 (17)	44.1 (156)
*Born in Sweden*						
Yes (1778)	12.8 (228)	8.2 (145)	8.0 (143)	12.4 (220)*	4.8 (86)*	53.8 (956)*
No (226)	18.1 (41)*	10.6 (24)*	11.9 (27)*	9.7 (22)	2.2 (5)	47.3 (107)
*Household composition*						
Living with partner (1181)	13.0 (154)	7.3 (86)	6.8 (80)	10.6 (125)	3.9 (46)	58.4 (690)
Living with parent (48)	10.4 (5)	16.7 (8)	6.2 (3)	10.4 (5)	4.2 (2)	52.1 (25)
Living with other (56)	14.3 (8)	5.4 (3)	7.1 (4)	10.7 (6)	7.1 (4)	55.4 (31)
Single (720)	14.3 (103)	10.0 (72)	11.5 (83)*	14.7 (106)*	5.4 (39)	44.0 (317)*
*Children in household*						
Yes (828)	12.7 (105)	9.3 (77)	7.6 (63)	11.1 (92)	3.3 (27)	56.0 (464)
No (1171)	14.1 (165)	7.9 (92)	9.1 (106)	12.7 (149)	5.5 (64)	50.8 (595)
*Education*						
Basic compulsory education or less (356)	22.8 (81)*	9.3 (33)	10.1 (36)	7.3 (26)	3.9 (14)	46.6 (166)
Secondary school (804)	10.9 (88)	7.8 (63)	7.6 (61)	12.6 (101)*	4.4 (35)*	56.7 (456)*
University or college (845)	12.0 (101)	8.6 (73)	8.6 (73)	13.6 (115)*	5.0 (42)*	52.2 (441)*
*Labour* *market* *position *						
Employment/self-employed/on leave/studies/parental leave (1638)	11.7 (191)	7.1 (116)	7.0 (115)	12.3 (201)	4.6 (76)	57.3 (939)*
Unemployment/labour market policy measures (93)	19.4 (18)*	10.8 (10)*	14.0 (13)*	12.9 (12)	4.3 (4)	38.7 (36)
Sick leave/disability pension (184)	21.7 (40)*	20.7 (38)*	17.9 (33)*	14.7 (27)	3.8 (7)	21.2 (39)
Retirement (70)	27.1 (19)*	4.3 (2)	7.1 (5)	1.4 (1)	4.3 (3)	55.7 (39)*
*Close friendship*						
Having a close friend (1804)	13.35 (243)	8.3 (150)	8.4 (151)	11.9 (214)	4.5 (81)	53.5 (965)
Not having a close friend (200)	13.5 (27)	9.5 (19)	9.5 (19)	13.5 (27)	5.0 (10)	49.0 (98)
*Somatic illness*						
Currently treated (853)	20.0 (171)*	10.9 (93)*	13.6 (116)*	9.6 (82)	5.2 (44)	40.7 (347)
Currently not treated (1152)	8.6 (99)	6.6 (76)	4.7 (54)	13.9 (160)*	4.1 (47)	62.2 (716)*
*Hazardous alcohol use *						
Yes (551)	12.0 (66)	11.3 (62)*	9.1 (50)	12.2 (67)	3.6 (20)	51.9 (286)
No (1279)	13.8 (176)	6.9 (88)	7.7 (98)	11.6 (148)	4.7 (60)	55.4 (709)
*Depression severity*						
Minor depression (172)	13.2 (38)	4.5 (13)	5.2 (15)	9.4 (27)	4.9 (14)	62.7 (180)
Major depression (287)	15.1 (26)	8.1 (14)	8.1 (14)	11.6 (20)	2.9 (5)	54.1 (93)
*Disability last 30 days due to psychological symptoms*						
Yes (428)	16.1 (69)*	13.1 (56)*	18.5 (79)*	15.4 (66)*	5.4 (23)	31.5 (135)
No (1568)	12.7 (199)	7.2 (113)	5.8 (91)	11.0 (173)	4.3 (68)	58.9 (924)*

*Showing significant differences.
